# Advances in new target molecules against schistosomiasis: A comprehensive discussion of physiological structure and nutrient intake

**DOI:** 10.1371/journal.ppat.1011498

**Published:** 2023-07-27

**Authors:** Peng Zhu, Kaijuan Wu, Chaobin Zhang, Syeda Sundas Batool, Anqiao Li, Zheng Yu, Jing Huang

**Affiliations:** 1 Department of Parasitology, School of Basic Medical Science, Central South University, Changsha, China; 2 XiangYa School of Medicine, Central South University, Changsha, Hunan, China; 3 Department of Microbiology, School of Basic Medical Science, Central South University, Changsha, Hunan, China; 4 China-Africa Research Center of Infectious Diseases, Central South University, Changsha, China; University of Bern: Universitat Bern, SWITZERLAND

## Abstract

Schistosomiasis, a severe parasitic disease, is primarily caused by *Schistosoma mansoni*, *Schistosoma japonicum*, or *Schistosoma haematobium*. Currently, praziquantel is the only recommended drug for human schistosome infection. However, the lack of efficacy of praziquantel against juvenile worms and concerns about the emergence of drug resistance are driving forces behind the research for an alternative medication. Schistosomes are obligatory parasites that survive on nutrients obtained from their host. The ability of nutrient uptake depends on their physiological structure. In short, the formation and maintenance of the structure and nutrient supply are mutually reinforcing and interdependent. In this review, we focus on the structural features of the tegument, esophagus, and intestine of schistosomes and their roles in nutrient acquisition. Moreover, we introduce the significance and modes of glucose, lipids, proteins, and amino acids intake in schistosomes. We linked the schistosome structure and nutrient supply, introduced the currently emerging targets, and analyzed the current bottlenecks in the research and development of drugs and vaccines, in the hope of providing new strategies for the prevention and control of schistosomiasis.

## 1. Introduction

Schistosomiasis is a major infectious disease worldwide that greatly endangers human health. It is estimated to affect more than 250 million people and cause approximately 280,000 deaths annually [1]. *Schistosoma mansoni* (*S*. *mansoni*), *Schistosoma japonicum* (*S*. *japonicum*), and *Schistosoma haematobium* (*S*. *haematobium*) are the major species that infect humans [2]. Notably, different species of schistosomes can cause distinct symptoms and negative effects on multiple human systems. Exposure to infested water is the primary cause of schistosome infection [3]. The cercaria in the water can rapidly penetrate human skin and then transform into juvenile schistosome, namely schistosomula, which migrate through the vascular system until they reach the most suitable blood vessel for survival. Preferences for blood vessels vary among schistosomes that accounts for difference of symptoms. Clinically, *S*. *mansoni* and *S*. *japonicum* mainly cause gastrointestinal and hepatosplenic disorders, such as abdominal pain, enteritis, and hepatosplenomegaly. On the other hand, *S*. *haematobium* is associated with genitourinary disorders, including hematuria, renal failure, squamous cell carcinoma of the bladder, and organ lesions of the prostate, seminal vesicles, or cervix [4–6]. In humans, female and male schistosomes develop and pair up as a prerequisite for egg production. Adult schistosomes living in the venous mesenteric, vesical plexuses, or other blood vessels lay hundreds of eggs daily. The deposited eggs are the main cause of the pathological manifestations in humans, leading to granulomatous reactions and subsequent chronic inflammation and fibrosis [6,7].

Anti-schistosomal drugs have been continuously developed, but most have been eliminated due to significant side effects, high costs, or other reasons. For example, hycanthone was effective in killing *S*. *mansoni* and *S*. *haematobium*, but its hepatotoxicity and mutagenicity led to its elimination [1]. The development of praziquantel (PZQ) is the greatest advancement in the field of schistosomiasis treatment. Currently, PZQ is currently the drug of choice because it is not only effective in killing the 3 aforementioned schistosomes, but also has good pharmacological properties and low cost [8]. PZQ, on the other hand, has been shown to be inefficient in killing schistosomula, and its extensive use has been linked to parasite drug resistance [1,8,9]. Historically, vaccine administration has been ranked as the most cost-effective method of preventing diseases caused by pathogens [10]. However, there is no schistosome vaccine available for human use. Over the past few decades, more than 100 schistosome antigens have been identified, but only a few candidates have entered human clinical trials [9]. One of the main reasons for this phenomenon is the improper selection or limitation of the models used for evaluation.

The development of drugs and vaccines should be based on a thorough understanding of schistosome characteristics. Schistosomes are obligate parasites; they completely depend on their hosts for growth and reproduction. In contrast to most other parasites, schistosomes feed on host blood, obtaining nutrients such as red blood cells, plasma proteins, glucose, lipids, and amino acids through their digestive tract or tegument [11]. For example, adult schistosomes consume nearly 5 times their dry weight of glucose per day through glycolysis to meet their energy requirements [12,13]. When glucose access is blocked, the survival and oviposition of schistosome are compromised [14]. Additionally, lipids are also essential for their oviposition [15]. The presence of large amounts of triglyceride suggests that schistosomes value this substance [16]. Moreover, proteins, amino acids, and even vitamins are also crucial for the vital functions of schistosomes. Therefore, describing the morphology and function of the schistosome tegument, esophagus, and intestine, understanding the essential factors to form and maintain these structures, and identifying the key factors that ensure their nutrition supply could significantly advance the development of drugs and vaccines to combat schistosomiasis.

In this article, we review studies on the morphology and function of schistosome tegument and digestive tract, which will greatly contribute to our overall understanding of this parasite. We further discuss the mechanisms involved in the uptake and utilization of nutrients by schistosomes and highlight some emerging targets that can block or inhibit this process. We aim to provide a direction for in-depth research and development of effective clinical treatments for schistosomiasis.

## 2. The basis of nutrient intake

The structural integrity and functionality of schistosomes is a prerequisite and basis for obtaining nutrition from the host, the most important of which are the tegument and the digestive system, including the oral, esophagus, and intestine.

### 2.1. The tegument

Schistosomes have a special shell called tegument, which is actually a syncytium, covered with membranocalyx [17,18]. The tegument plays an important role in nutrient absorption, defense, osmotic pressure regulation, and excretion. As a key site of host–parasite interaction, the tegument has attracted extensive attention, and its changes have been used to assess the effects of anti-schistosomal drugs [19].

**Fig 1:**
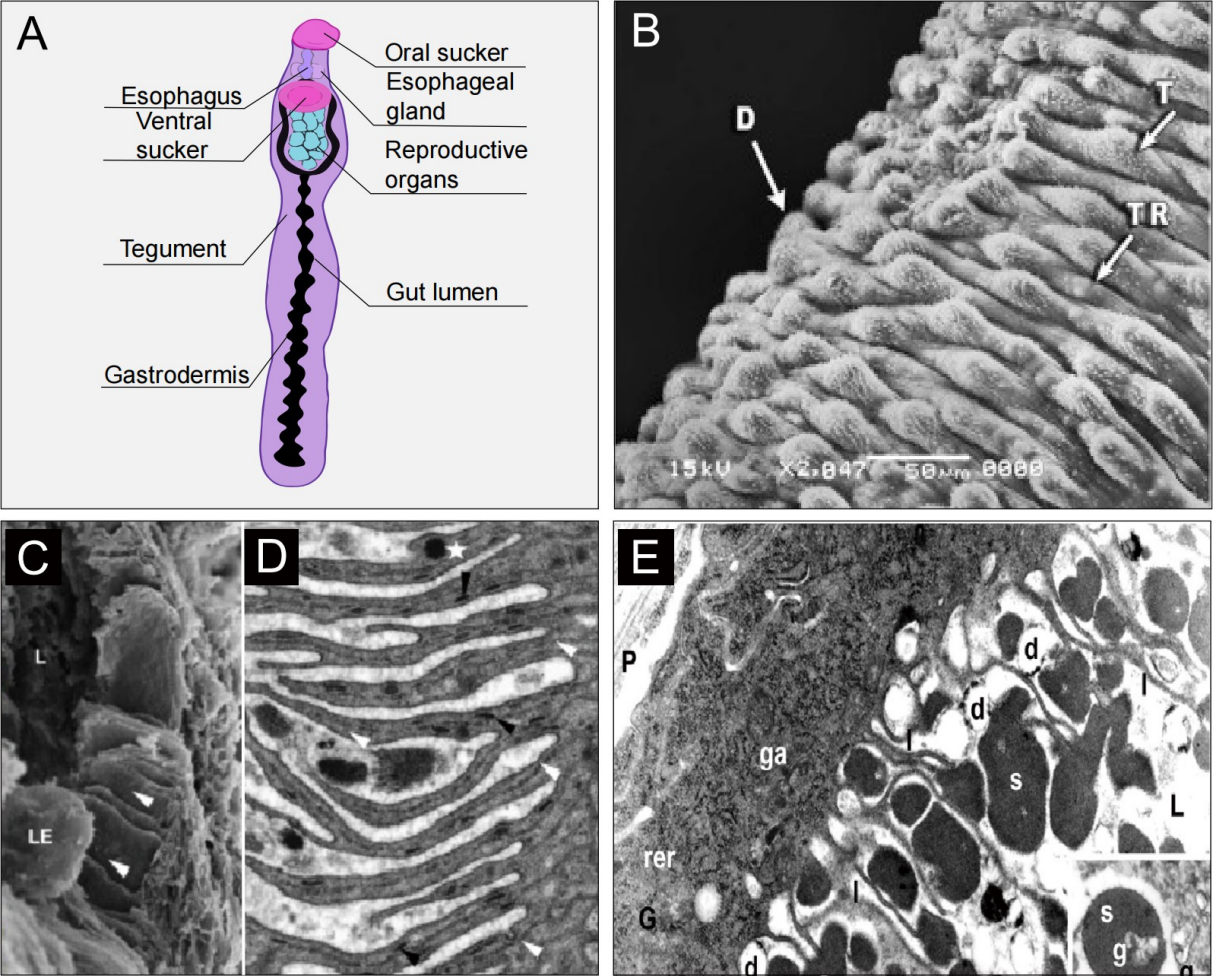
Morphology of schistosome. **(A)** The general morphological structure of schistosomes. **(B)** Adult male worm with well-developed tubercles (T) and tegumental ridges (TR) on the dorsal surface (D) (×2,047). Adapted from ref [23]. **(C)** SEM of the posterior esophageal lining of *S*. *mansoni* showing the luminal surface greatly extended to form thin plates. (L, Esophageal lumen; LE, Leucocyte.) **(D)** TEM of the thin plates. A central double line (white arrows) is evident in each plate and discoid bodies (black arrows) are numerous. Adapted from ref [32]. **(E)** TEM of the *S*. *mansoni* gut epithelium. The cytoplasm of the syncytial gastrodermal epithelium (ga) is rich in the rough endoplasmic reticulum (rer) and Golgi apparatus, typical of a cell synthesizing proteins and glycans for export. The luminal surface is extended by numerous thin lamellae (l) 3–5 microns long. (s, erythrocyte stroma; g, pigment granule; d, lipid droplets). Adapted from ref [11].

The structures of schistosome tegument in multiple life stages, including cercariae, schistosomula, adult worm, miracidia, and sporocyst, were described decades ago [20]. Fig 1A illustrates the general morphological characteristics of schistosome. The syncytial layer covers the entire surface of both male and female worms and extends in a highly modified form as the inner layer of the esophagus up to its junction with the gastrodermis [21]. Observation of *S*. *mansoni* cercariae by transmission electron microscopy revealed the presence of distinct spines on the tegument, as well as basement membranes, circumferential and longitudinal muscles beneath the tegument [22]. Most structures observed in cercariae are also present in both juvenile and adult worms, such as myofibrils, multilayered vesicles, and discoid bodies [21]. On the ventral surface of the 3-week-old juvenile *S*. *mansoni*, oral and ventral suckers and a bore exhibiting the beginning of the gynecophoral canal can be observed, while many folds can be seen on the dorsal surface [23]. In adult males, oral and ventral suckers, gynecophoral canal, and well-developed tubercles and tegumental ridges can be found [23] (Fig 1B). Schistosomes attach to the vascular wall with suckers, and these folds increase the contact area between the worm body and the host blood, which is conducive to nutrient intake.

The presence of multiple functional proteins on the tegument also confirms its important role in nutrient acquisition [24]. So far, several dozen major proteins belonging to different protein families have been identified, including enzymes, structural proteins, and some schistosome-specific proteins whose functions are yet to be studied [25]. Among these proteins, some are involved in or assist in the transport of nutrients across the tegument [21,26,27]. For instance, schistosome glucose transporter protein 1 and 4 (SGTP1/4) contribute to glucose intake [28,29]. In addition, enzymes like calpain on the tegument contribute to degrading host blood clotting protein and impeding the formation of blood clots [30], ensuring that important structures on the tegument are not covered. Moreover, upon entry into the mammalian host, the tegument undergoes significant changes [31], suggesting that it would change in response to different conditions to improve the ability of nutrient intake.

### 2.2. The esophagus

Structurally, the schistosome esophagus is extremely complex and highly organized. In living adult schistosomes, the esophagus is linear and can be divided into anterior and posterior compartments, the former being about 1/3 as large as the latter, with a clear boundary between them [32]. Both chambers are surrounded by a mass of cell bodies and lined with a syncytial layer of cytoplasm continuous with the tegument [32,33]. It has been found that the cell masses of both anterior and posterior chambers have the function of synthesizing and secreting proteins [33–35], particularly the cell bodies of the posterior esophagus, which constitute the esophageal gland [32]. These cell bodies extend and pass through the esophagus muscle and are connected to the lining syncytium. In the confocal microscope (at the limit of resolution), the posterior lining exhibits plate-like shadowy stripes that are essentially thin cytoplasm tapering towards their tips [32]. The area of the posterior esophageal cavity is significantly increased due to the existence of these plate-like structures [32]. The plates also ensure the smooth entry of esophageal gland secretions into the esophagus lumen and their full contact and reaction with the ingested substance (Fig 1C and 1D).

As one of the most significant food sources for schistosomes, blood feeding is a multi-step and continuous process [32]. The initial step is a rapid grabbing motion of the oral sucker (4/sec), and blood passes through the open oral sphincter to accumulate in the anterior esophageal lumen. In the second step, peristaltic movements push the food ball into the posterior esophagus. The passage of ingested blood into the transverse gut, which is likely to occur when the posterior sphincter opens, is the last step. Food scraps are finally ejected from the oral route due to vigorous activity of the gut and relaxation of the esophageal wall. There is a notable difference between blood intake of adult male and female parasites. Specifically, the female consumes some 330,000 red blood cells and 20 nl plasma per hour, which is 8 to 10 times that of male worms [11], suggesting that blocking blood access to females may be more effective.

The esophagus is also a processing site for various blood components such as erythrocytes, leucocytes, and antibodies. Intact erythrocytes were found in anterior esophagus lumen [36], but rarely in the intestinal lumen, suggesting that the vast majority of erythrocytes are rapidly subjected to early processing in the posterior esophagus [32]. A proteomic analysis of worm vomitus found that erythrocytes are deshelled upon passage through the esophagus and interacted with esophageal secretions [37]. In addition to erythrocytes, bound leukocytes can also be observed in the posterior esophagus, which are damaged and destroyed to varying degrees [32]. Studies based on cytology and gene expression also confirmed that the esophageal gland is the site of the initial processing of ingested blood cells [11,32,33,38].

### 2.3. The intestine

The intestine continues with the esophagus, bifurcates posteriorly at the ventral sucker, and extends on either side of the reproductive organs, before joining to form a single part that continues to the posterior end of the worm. The surface of the intestine is called gastrodermis, which is syncytial like the tegument but differs in the presence of numerous mitochondria, active Golgi apparatus, and rough endoplasmic reticulum [11,39,40]. Consequently, the gastrodermis has a stronger ability to synthesize and export proteins. The surface of the intestinal lumen is covered with many cytoplasmic lamellae ranging in size from 3 to 5 μm and performing absorptive functions similar to traditional microvilli [11,41] (Fig 1E).

The intestine is the site of digestion and absorption of ingested substances, especially hemoglobin and plasma proteins [37,42]. The action of several enzymes is crucial for normal functioning of the intestinal tract. The collection of schistosome vomit is one of the currently feasible methods to study its intestinal proteomics, by which the digestion of host hemoglobin and plasma proteins was confirmed [43]. Several studies confirmed the presence of proteases in the gastrodermis by immunocytochemistry and pointed out their role in host protein processing and degradation [44–47]. In line with the results of proteomic studies, gene expression profiles revealed an abundance of transcripts encoding a range of endo- and exo-peptidases in the gastrodermis [48,49].

Multiple enzymes in the worm gut formed a complex enzyme network that is sequentially involved in the catabolic digestion of host hemoglobin, plasma proteins, and other substances. For example, saposin-like proteins have been identified in gastrodermis, and they could interact with lipids on cell membranes [37,50]. In other words, saposin-like proteins may be involved in the acquisition of host lipids from serum and transport into schistosome cells. *S*. *mansoni* cathepsin L1 (SmCL1) could degrade human hemoglobin into dipeptides and amino acids [51]. And the suppression of aspartate protease, *S*. *mansoni* cathepsin D (SmCD), would result in the failure of schistosome to develop and survive in mice [47]. In addition, antioxidant enzymes such as glutathione peroxidase and superoxide dismutase are present in the schistosome intestine and tegument, which protect the worms from hemoglobin’s oxidation products and host cell responses [52,53]. Notably, the transcription levels of most antioxidant enzymes such as Cu–Zn superoxide dismutase and glutathione peroxidase are significantly lower in juveniles contrary to adults [52], suggesting that adults have a relatively high requirement for nutrient and that targeted suppression of antioxidant enzymes kills adults more effectively than schistosomula. The key proteins identified so far in schistosome intestine are summarized in Table 1, along with their functions and inhibitors.

**Table 1:** Enzymes in the intestinal tract of schistosome and their functions and inhibitors.

Protein	Function	Inhibitor	Reference
Cathepsin B1 (SmCB1/Sm31)	Degrades hemoglobin α and β chains; degrades the plasma proteins such as albumin, IgG, and α-2 macroglobulin	E-64 and CA-074 methyl ester	[111,131,132]
Cathepsin L1 (SmCL1/SmCF)	Degrades hemoglobin	Peptidyl fluoromethyl ketones, vinyl sulphones, and epoxysuccinyl derivatives	[133–136]
SmCL2	Might contribute to ingested protein degradation	Cystatins	[44,132,137,138]
SmCL3	Hydrolyzes albumin and hemoglobin	E-64 and K11777	[139,140]
Asparaginyl endopeptidase (SmAE/legumain /Sm32)	Acts as a protein processor, can trans-activate SmCB1 and SmCL1 to their mature catalytic forms	Aza-peptide Michael acceptors and epoxide inhibitors	[111,141–143]
Cathepsin D (SmCD/aspartic protease)	Plays an apical role in the digestion of hemoglobin	Antibodies generated to lipid core peptides	[47,144,145]
Superoxide dismutase	Resists oxidation products and host cell responses	Diethyldithiocarbamate and tetrathiomolybdate	[52,53,146]
Saposin-like proteins	Promotes lipid degradation and absorption	Unknown	[37,50]

## 3. Mechanism of nutrient intake and utilization

### 3.1. Glucose

Carbohydrate metabolism is the most important physiological process of schistosomes in the host. Glucose is the primary source of energy for schistosomes, providing the large amount of the energy required for worm growth and reproduction, making it one of the most important nutrients [13,54]. During the complex life cycle of schistosomes, the alternation between the consumption of their stored glycogen and host glucose is necessary to meet the energy requirements. After entering the mammalian host, the metabolic characteristics of cercariae will change dramatically, including the reversible change from oxidative metabolism to glycolytic metabolism depending on glucose concentration [55,56]. It was found that adult schistosomes consume up to 20% of their dry weight of glucose per hour, with glycolysis as the primary metabolic pathway [57]. Glucose interruptions, it turns out, do affect the survival of worms [14]. The mechanisms by which schistosomes obtain glucose and the factors that may affect glucose intake or regulate metabolism have been investigated to find potential intervention strategies.

Schistosomes may rely primarily on glucose transporters for glucose acquisition. Schistosome glucose transporter protein 1 (SGTP1) and SGTP4 play a major role in glucose intake [58]. Both SGTP1 and SGTP4 are expressed in *S*. *japonicum* and *S*. *mansoni*; however, there is no report on the expression of SGTP4 in *S*. *haematobium* [59,60]. SGTP1 is distributed in the basement membrane and internal tissues and is expressed in the eggs, sporocyst, cercariae, schistosomula, and adult worms; whereas SGTP4 appears to be located only in the apical membrane of mammalian-stage parasites [29,58]. The distribution characteristic means that SGTP4, which interacts with the host, is responsible for obtaining glucose from the host blood, while SGTP1 transports free glucose into the interstitial fluids [28]. In both juvenile and adult worms, the suppression of SGTP1 or SGTP4 will limit glucose intake, and a significantly greater impairment is exhibited with both genes suppressed [14]. SGTP-suppressed worms have significantly reduced viability in vivo, which further confirms the pathway and importance of glucose. To maximize glucose uptake during glucose deprivation, the parasite may increase the levels of SGTP1 and SGTP4 [55].

The glucose transporters are indispensable in glucose uptake, but we also note that there are multiple signaling pathways that may regulate this process. According to a new study, Akt (also known as protein kinase B) is required for SGTP4 expression, and Akt inhibition could reduce worm glucose uptake [61]. The role of insulin in facilitating glucose transport by glucose carriers is well established, and the insulin pathway in schistosomes is similar to that of other organisms [62]. Schistosome insulin receptors (IRs), located in the tegument, belong to the large class of receptor tyrosine kinases and can bind to human insulin [63,64]. These IRs can activate downstream signaling transduction of tyrosine kinases to regulate glucose uptake after binding to insulin [65,66]. Mechanistically, insulin signaling pathway can activate the Akt signal in worms, which in turn promotes the expression of glucose transporter proteins [61]. Taking *S*. *japonicum* insulin receptor 1/2 as an example, it has been confirmed that their suppression can significantly reduce glucose intake, in turn negatively affecting parasitic growth and development [66]. Studies have identified the site where IR1/2 binds to host insulin, supporting the design of vaccines [67]. It should be noted that, in addition to host insulin, schistosome IRs can also bind to the schistosome insulin-like peptide, thereby activating the worm’s insulin pathway [68]. Further studies confirmed that vesicle-associated membrane protein 2 (VAMP2) knockdown significantly affected the transcript levels of *SGTP1*, *SGTP4*, and insulin receptors (*IR1* and *IR2*) [69]. In female worms, *vamp2* RNAi mainly caused a reduction in egg production [69], which may be influenced by impaired energy access in males [70].

Schistosomes are capable of aerobic glucose metabolism, but glycolysis is their primary mode of energy acquisition in hosts. A number of key enzymes involved in the schistosome glycolytic pathway have been identified, such as triose-phosphate isomerize [71] and glyceraldehyde-3P-dehydrogenase [72]. Recent studies revealed that AMP-activated protein kinase (AMPK) plays a significant role in the regulation of larval viability and adult sugar metabolism [12]. AMPK is conserved in all eukaryotes and is the main regulator of intracellular energy metabolism and energy homeostasis [73,74]. AMPK may not be essential for adults, but it is certain that it regulates adult worm glycogen synthesis, storage and utilization, ensuring the survival of schistosomes in situations such as transient glucose deprivation [12]. Fructose-1,6-bisphosphate aldolase (FBPA) plays a key role in glycolysis by converting fructose-1,6-biphosphate into dihydroxyacetone phosphate and glyceraldehyde phosphate. It was suggested that *S*. *japonicum* FBPA may enable the worm to obtain more metabolites and win the nutrient competition with the host, due to its lower K_m_ and higher enzymatic efficiency for degrading fructose-1,6-biphosphate than host FBPA [75]. Unfortunately, the expression of FBPA in *S*. *mansoni* and *S*. *haematobium* has not been reported.

As glucose is primary source of energy for schistosome, hence, current research focuses on mode of glucose uptake used by parasite. Glucose intake can be limited or blocked by inhibiting the expression of related molecules, blocking receptor–ligand interactions, or destroying the morphological structure and function of the tegument.

### 3.2. Lipids

Lipids account for at least 1/4 of the dry weight of adult schistosomes, and worms must obtain lipids from external sources to meet their needs [11]. It was found that males consume only 2.5% of their lipid content per day, while females consume up to 50%, suggesting that males have a higher lipid storage capacity or females possess a higher ability to utilize or excrete lipids [11]. Human plasma lipids include triglycerides, phospholipids, cholesterol and its esters, as well as free fatty acids. The current understanding of the mechanism of lipid acquisition and utilization by schistosomes is limited, and we speculate that plasma lipids and erythrocyte membranes may be the main source.

Fatty acid is one of the essential lipids for schistosomes. The fatty acid-binding protein family member of *S*. *mansoni* called Sm14 may play an important role in fatty acid acquisition [76]. Fatty acid oxidation (FAO) has been reported to be critical for schistosome oviposition [77,78]. It was found that inhibition of carnitine palmitoyl transferase 1, which catalyzes the rate-limiting step of FAO, and loss of acyl CoA synthetase or acyl CoA dehydrogenase function would significantly decrease the rate of oxygen consumption and egg production of schistosomes [77]. But some researchers have doubts about the idea because no direct evidence of FAO has been observed in schistosomes, and the genes coding for FAO-related enzymes are absent in *S*. *mansoni* genome [79]. Michiel and colleagues used ^14^C-labeled fatty acids to study their metabolism in adult *S*. *mansoni* worm pairs, and the results showed that adult worms have a limited ability to metabolize lipids, despite that they have a need to acquire fatty acids [79]. In any case, different studies point to the absolute necessity of fatty acids for schistosome spawning. Of note, arachidonic acid, a kind of fatty acid, was identified as an endoschistosomicide [80].

Some tegument lipids have been found to modulate host immunity, thus keeping themselves safe from excessive damage. For example, lyso-phospholipids are abundant in *S*. *mansoni* tegument, mainly eicosenoic acid (20:1)-containing lyso-phosphatidylserine and lyso-phosphatidylethanolamine species [81]. It has been proposed that lyso-phosphatidylserine can act on host dendritic cells, which differentiate T cells towards the Th2 and Treg phenotypes [82]. When incubated with linoleic acid, schistosomes secrete eicosanoids, including hydroxyeicosatetraenoic acid (HETE), prostaglandin E2 (PGE2), and prostaglandin D2 (PGD2), which suppress host immunity and benefit worm survival [83,84].

In general, lipids are one of the essential nutrients for schistosomes and are mainly used for biosynthesis that ensures the worm’s oviposition and resistance to host immunity.

### 3.3. Protein and amino acids

Protein serves as the foundation for schistosome life activities, which is essential for nutrient uptake, structural formation and maintenance, and the interaction between worms and hosts. Recently, schistosome tegumental ectoenzymes were found to play a key role in the regulation of host immunity, which facilitates the long-term survival of the worm in the host [82]. Adult worms express multiple enzymes on their surface that cleave important immune signaling molecules in the blood, potentially impairing their ability to activate anti-parasitic immune cells. In addition, the worms release a slew of proteins that directly interact with host cells to suppress their activation [82].

It is estimated that adult male and female *S*. *mansoni* consume approximately 39,000 and 330,000 red blood cells per hour, respectively [85], and plasma proteins are also the main components obtained from host. The uptake and processing of erythrocytes or proteins involve the oral parts, esophagus, and intestinal tract and their secretions. The processing of ingested proteins is mainly the responsibility of a complex network system composed of multiple proteases, which has been briefly described previously. For example, inhibition of SmCB1 leads to the slow growth of parasites [86]. The inhibition of SmCD may lead to the inability of schistosomes to digest hemoglobin normally, and they will not survive to maturity after infecting mice [47].

Different from the way of protein acquisition, schistosomes can obtain the amino acids through the tegument. Early studies have shown the existence of several different amino acid uptake systems in adult *S*. *mansoni* [87]. As a member of the glycoprotein-associated transporter family, the schistosome permease 1 (SPRM1) light chain, is characterized in *S*. *mansoni*, which is present in multiple tissues of juvenile and adult worms [26]. SPRM1 heavy chain is also expressed in all stages of schistosome life [88]. The expression of SPRM1, composed of SPRM1 light chain and heavy chain, in *Xenopus Oocytes* can increase the uptake of various amino acids, such as histidine, arginine, lysine, leucine, phenylalanine, methionine, glutamine, and tryptophan [88]. The expression of SPRM1 in the tegument of schistosomes at various life stages shows the significance of amino acids to their life activities. Taking methionine as an example, the methionine bound by unpaired females is only 30% of that of paired females, which indicates that females need more amino acids to maintain metabolism during mating with males [89]. The use of recombinant human TNF-α can lead to reduced methionine uptake resulting in impairment of reproductive capacity [89].

**Fig 2:**
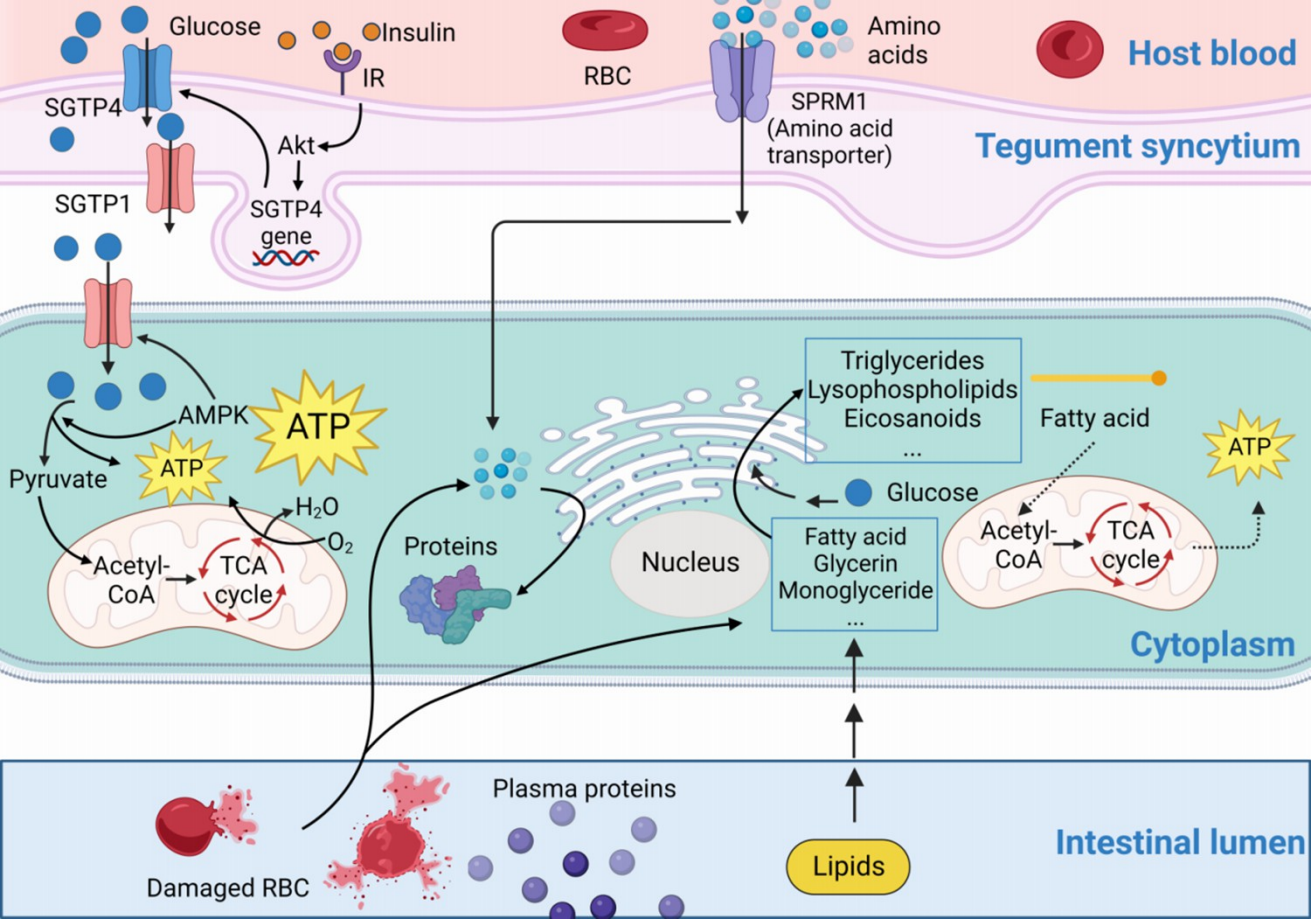
Intake and utilization of glucose, lipids, protein, and amino acids by schistosome. Schistosomes obtain glucose, lipids, proteins, and amino acids from host blood for energy production and as substrates for the synthesis of their own substances to ensure normal life activities such as growth, reproduction, and egg production. The dotted line represents controversial or uncertain content at present. Created with BioRender.com.

The uptake and utilization of glucose, lipids, proteins, and amino acids are shown in Fig 2. Besides, other substances also have significant effects on schistosomes. For example, there are high expressions of enzymes involved in vitamin B6 metabolism in both juveniles and adults [90], which indicates that vitamin B6 drives a series of metabolism in schistosomes. Both, the specificity of pyrimidine transport and active metabolism suggest its importance in schistosomes [91].

Interestingly, schistosomes can cause metabolic alterations in their human host that facilitates their predation of nutrients. It was found that metabolite profiles associated with host energy and purine metabolism changed significantly after a period of *S*. *haematobium* infection [92]. The parasite’s demand for energy and other substances increases host glycogenolysis, gluconeogenesis, and fatty acid synthesis, stimulates host glycolysis, and leads to increased levels of host ADP, AMP, G6P, and 3-PG, which correlate positively with the intensity of infection [92,93]. In addition, tegumental enzymes dephosphorylate host G6P, AMP, and ADP, facilitating parasite in uptake of glucose and adenosine [94]. The plunder of host energy leads to alterations in host metabolism, which in turn provides additional nutrients to schistosomes to some extent. We believe that all infectious species of schistosomes cause alteration in host metabolism, and blocking or reducing the nutrient intake by schistosomes is not only damaging to the parasite but also reduces the degree of alteration in host metabolism, which may be a positive feedback process.

In summary, the uptake of nutrients by schistosomes, whether through the tegument or the digestive tract, is based on structure and various types of molecules. These nutrients not only provide energy or substrates required for anabolism, but are also essential for maintaining the structure and affecting host immunity. The importance of clarifying the mechanisms of nutrient uptake and utilization for the elimination of schistosomes in the body cannot be overstated.

## 4. Emerging potential targets

Based on the understanding of how schistosomes obtain nutrients, here we will present several emerging targets that are expected to help us disrupt their structure and nutrient intake. The functions of these key molecules are shown in Table 2.

**Table 2:** The distribution and function of some important proteins and the effect after being inhibited.

Target	Main distribution	Possible functions	Effect of being suppressed	Reference
VAMP2	Tegument (*S*. *japonicum*)	Mediates membrane fusion and maintains normal tegument morphology	Tegument shedding and the oversized bulb-like structure formation	[69,97]
TSP-2	Tegument (*S*.*mansoni*, *S*. *japonicum*, and *S*. *haematobium*)	Acts as a scaffold for protein complex formation	The tegument becomes thinner and forms vacuoles	[101–103,147]
MEG-4.1	Esophagus (*S*.*mansoni* and *S*. *japonicum*)	Involves in the processing of host cells	May not be able to escape the host immune response	[32,107]
FoxA	Esophagus (*S*.*mansoni*)	Maintains MEG-4.1 expression and ensures the esophageal gland cell differentiation	Ablation of the esophageal gland	[110]
FTZ-F1 and MEG-8.3	Esophagus (*S*.*mansoni*)	Maintains the integrity of the esophageal gland and head	Progressively lose the ability to accumulate nutrient	[108]
HNF4	Intestine (*S*.*mansoni*, *S*. *japonicum*, and *S*. *haematobium*)	Contributes to the renewal of intestinal stem cells and the formation of microvilli-like structures	Reduced expression of gut markers, impairment of gut production, and feeding disorders	[114–116,148]

### 4.1. Targeting the tegument

As the site of nutrient acquisition and direct contact with the host, the tegument plays an important role in schistosome survival. In our opinion, molecules required for the development and maintenance of the tegument, as well as for proper functioning, are potential anthelmintic targets.

Hitherto, many experimental results have proved our point. The tegument is not a static structure and it can continuously absorb new cells produced by stem cells, which facilitates the maintenance of the structure and function, as well as evasion of surveillance by the host immune system [95]. Many drugs or compounds can cause high mortality and significant changes in the tegument. For instance, when exposed to PZQ or primaquine, both juvenile and adult worms show erosion, peeling, and sloughing of the tegument, as well as extensive damage to the subtegumental layers [23].

**Fig 3:**
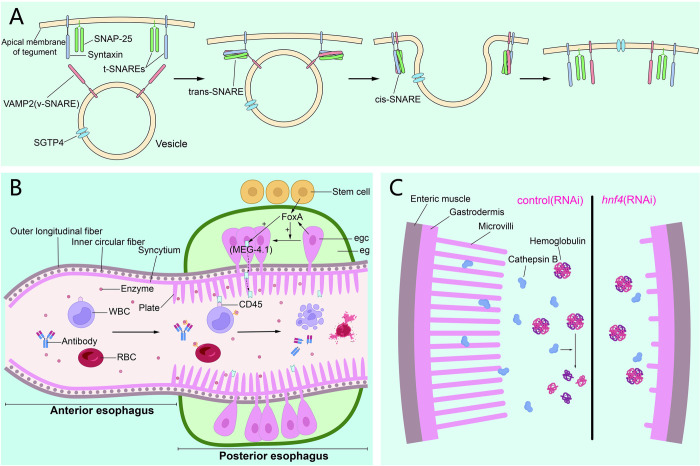
Functions of VAMP2, FoxA, and HNF4 in schistosome. **(A)** VAMP2 on the vesicle membrane pairs with t-SNAREs on the target membrane to form a complex that drives membrane fusion for maintaining the stability of the apical tegument membrane and molecular transport, and to transfer SGTP4 on the vesicle membrane to the tegument. **(B)** Esophageal gland plays an important role in the digestion of blood components such as B cells, leukocytes, erythrocytes, and antibodies. FoxA expressed by esophageal gland cells (egc) and stem cells neighboring esophageal gland (eg) can promote the differentiation and development of esophageal gland cells and the expression of MEG-4.1, which binds cells by binding to molecules on the surface of leucocytes. **(C)** Schematic diagram of the influence of *hnf4*(RNAi) on the intestinal tract of schistosome. (SNARE, soluble N-ethylmaleimide sensitive factor attachment protein receptor; v-SNARE, VAMP2 on the vesicle membrane; SNAP-25, synaptosomal-associated protein of 25 kDa; t-SNARE, syntaxin and SNAP25 on the target membrane).

Biological membrane fusion is a key step in processes such as cell growth, and it is also an essential mechanism to control the transport of cargo molecules [96]. The soluble N-ethylmaleimide-sensitive factor attachment protein receptor is required for membrane fusion, and within this family, vesicle-associated membrane protein 2 (VAMP2) is an important member. Studies have found that VAMP2 is involved in the maintenance of the schistosome tegument morphology [97], although the current report is only about *S*. *japonicum*. The *Vamp2* gene is mainly expressed in the tegument and its transcript is abundantly expressed in schistosomula and adult worms [97]. Suppression of *Vamp2* revealed that 67% of the treated worms had significant changes in their tegument and underlying tissues, manifested as partial tegument shedding, and the formation of oversized bulb-like structures [69] (Fig 3A).

Recently, it has been found that the intermediate cells generated by stem cells express zinc finger proteins, and the cells that constitute the tegument express a protein called tetraspanin 2 (TSP-2), which are required for the structural formation of tegument [95]. Proteins with zinc finger motifs typically participate in regulatory processes and are thought to be the most important class of transcription factors in eukaryotes. In the nucleus of adult male worms, cercariae, and schistosomula cells, zinc finger protein 1 was detected and was shown to be a transcription factor [98]. The function of the TSP protein in schistosomes is currently unknown; it may be involved in the formation of protein complexes by acting as a scaffold, and these complexes can promote vesicle fusion or fission [99,100]. After the silencing of *Sm-tsp-2*, the tegument of the schistosomula and adult worms becomes thinner and more vacuolated, and the survival ability of worms after treatment decreases dramatically [101,102].

As a significant part of schistosomes, the normal tegument function is crucial to the survival of the worm, and of course, it is also our target to kill schistosomes. *S*. *mansoni*, *S*. *japonicum*, and *S*. *haematobium* have no obvious difference in the morphological characteristics of the tegument and even some proteins have high homology, such as Sm-TSP-2, Sj-TSP-2, and Sh-TSP-2 [103,104]. It implies that drugs or vaccines designed for certain molecules of the tegument may have similar killing effects on the 3 species, which greatly simplifies drug or vaccine development.

### 4.2. Targeting the esophagus

Studies on the secretions, development, and maintenance of the esophagus are expected to help expedite the development of promising vaccine and drug candidates or other strategies for schistosomiasis therapy.

It has been proposed that a mixture of approximately 40 proteins specifically secreted by the esophageal glands is responsible for initiating blood processing in the esophagus [35]. Currently, some microexon gene (MEG) products and venom allergen-like proteins have been found to be esophageal gland-specific proteins [32,105,106], which may play a role in capturing or lysis of host cells. There are 13 proteins encoded by MEGs in the male schistosome esophagus, 11 out of them are uniquely located in the esophageal glands. As an example, in *S*. *mansoni* and *S*. *japonicum*, the MEG-4.1 protein, the main secretory product of the glandular cells, is expressed exclusively in the esophageal gland [32]. MEG-4.1 is O-glycosylated, which makes it strongly adhesive so that it can be distributed over the entire esophageal surface [32,107]. Furthermore, MEG-4.1 can be bound to pan-leucocyte markers such as CD45 to target leukocytes [32] and may be involved in capturing and destroying leukocytes.

Similarly, *Meg-8*.*3* is only found in the esophageal gland and is required for its maintenance as well as the integrity of the head [108]. As expected, *meg-8*.*3* knockout worms progressively lost their ability to accumulate nutrients in the intestine [108]. In *S*. *mansoni*, the transcriptional regulator protein fushi tarazu-factor 1 (FTZ-F1) was found to maintain the integrity of the esophageal gland and head through controlling *meg-8*.*3* expression, the loss of either will lead to head tissue degeneration and esophageal gland dysfunction [108].

Forkhead box protein A (FoxA) has been shown to play a role in the regeneration of the pharynx in turbellaria [109]. In the esophageal gland of juvenile and adult schistosomes, *foxA* was found to be as highly enriched as *meg4*.*1*, and most of the cells expressing *foxA* also expressed *meg4*.*1*. Moreover, *foxA* expression was also found in adjacent stem cells [110]. In *foxA* RNAi schistosomula, *meg4*.*1* expression was not detected and differentiated gland cells disappeared in the esophageal gland region and were replaced by the stem and progenitor cells [110]. This suggests that *foxA* is necessary for the differentiation of esophageal gland cells and, consequently, for the development of the esophageal gland. The esophageal gland of adult worms is ablated after *foxA* knockout but has no effect on worm’s morphology and behavior in vitro [110]. Nevertheless, significance of esophageal gland for adult schistosomes in host is inevitable. This is confirmed experimentally: in mice, *foxA* RNAi schistosomes cause significantly fewer liver granulomas compared to controls, and dead worms can be found in the liver sinusoids; even if *foxA* RNAi parasites are fortunate enough to survive in vivo, their size is significantly reduced [110]. In addition, schistosomes lacking esophageal gland have abnormal esophageal degradation of ingested immune cells, cannot prevent immune cells from entering the intestine, and are more likely to be killed by host immune cells in mice [110] (Fig 3B).

Overall, the schistosome esophagus is essential for feeding; its development and maintenance, as well as food processing need the synergistic action of a number of molecules. Inhibiting any of the critical component expressions involved in aforementioned processes would be lethal to the parasite.

### 4.3. Targeting the intestine

In schistosomes, the key site for processing of ingested substances is the intestine, where the complex enzyme network is a hot spot for drugs and vaccines research. Additionally, disrupting the formation and maintenance of its structure is equally relevant.

A variety of proteins in the intestinal tract of schistosomes are indispensable for digesting, which provides a direction for vaccine and drug design [38]. For instance, *S*. *mansoni* cathepsin B1 (SmCB1) is the most abundant papain-like cysteine peptidase in the gastrodermis, which was first located in the gut lumen [111]. It was found that SmCB1 is the specific target of IgG and IgE [112], and subcutaneous injection of SmCB1 with functional activity has a significant protective effect on worm infection [113]. Further study found that cysteine peptidase has the ability to act as both an immunogen and adjuvant, and it has a stronger protective effect when combined with other vaccine candidates [113].

Hepatocyte nuclear factor 4 (HNF4) is a fatty acid-binding transcription factor that plays an important role in the development and maintenance of schistosome intestinal tract. HNF4 is a marker of turbellaria gut neoblasts [114], and *hnf4* knockdown results in reduced expression of gut markers and impairment of gut production [115]. Transmission electron microscopy reveals that *hnf4* RNAi schistosome intestinal tissues, although present, have significantly fewer microvillous-like structures in the lumen (Fig 3C). In terms of uptake and digestion of erythrocytes, most *hnf4* RNAi schistosomes failed to uptake or digest erythrocytes. In addition, proteolytic enzyme expression was reduced in these schistosomes, and cathepsin B activity was significantly lower [115]. Infection experiments revealed normal liver morphology in mice receiving the *hnf4* RNAi parasite, whereas a large number of egg-induced granulomas were present in control group [115]. However, the role of HNF4 in the development and maintenance of the schistosome gut remains unclear. In addition, it has been demonstrated that HNF4 is an important conserved transcription factor for the brush border gene program in organs including the intestine [116]. Loss of HNF4 in the mouse intestinal epithelium will result in severe disruption of the brush border [116], and this may also be one of the reasons for the apparent reduction of microvillus-like structures in *hnf4* RNAi schistosomes.

Overall, the structural formation and maintenance of the schistosome tegument, esophagus, and intestine, as well as their normal function, provide us with many potential targets. Structural destruction of schistosomes will undoubtedly affect their nutrient uptake, which in turn will affect the structure and viability of the worm.

## 5. Conclusion and perspectives

Schistosomiasis is a prevalent parasitic disease worldwide, with its public health and socioeconomic impact growing exponentially due to population movement factors such as migration and international tourism [117]. Treatment of schistosomiasis has primarily relied on the single use of PZQ in the past decades [118]. However, we cannot help but worry about how long the effectiveness of this drug will last without a viable alternative or combination strategy. Therefore, there is an urgent need to change this situation to avoid the emergence of drug-resistant strains in clinical treatment [119].

Blocking the nutrient supply of schistosomes and destroying their structure can possibly be imagined as a consensus treatment for schistosomiasis. The morphological characteristics of *S*. *mansoni* in malnourished mice were studied at the beginning of 21st century. It was shown that the nutritional status of the host does have a negative impact on schistosomes, such as a significant reduction in body length and width, ovarian length, and area of testicular lobes [15]. Moreover, nutrient-deficient schistosomes are less likely to evade host immune system and are less resistant to drugs. In order to obtain sufficient and various forms of nutrients from the host, schistosomes form a complex feeding system in which the tegument and digestive tract act in concert. The structure, nutrient intake, and various life activities of schistosomes are linked, and destroying one of them will undoubtedly break this cycle. We believe that the choice of therapeutic strategies depends on the duration of the infection and the life cycle of the schistosomes in the patient. For example, in the early stage of infection, when we need to timely inhibit the growth and development of schistosome to reduce its viability, inhibition of glucose acquisition is particularly important.

Tegument, esophagus, and intestine are crucial to the development and functions of schistosome and can serve as breakthrough point for research. As mentioned previously, VAMP2 mediates membrane fusion and therefore may play an important role in the maintenance of tegument morphology as well as in component renewal [97]. In contrast, abnormal expression of FoxA and HNF4, which are involved in the development of the esophagus and intestine respectively, may also negatively affect esophageal and intestinal secretion [110,115]. By targeting these molecules, we can achieve a better killing effect by structural destruction and nutrient access disruption. It is feasible to design drugs or vaccines against these targets, but we cannot ignore the structural homology between the parasitic and human biomolecules to prevent harmful effects on host.

At present, anti-helminthic drugs and vaccines are being designed and developed based on these structures and related molecules. For instance, polypyridylruthenium (II) complexes can inhibit acetylcholinesterase activity in the tegument, significantly impair glucose uptake capacity, and are effective against schistosomula and adult schistosomes [120]. Back to the star drug against schistosomes, PZQ, its anti-helminthic effect may be related to muscle paralysis and tegument damage [121,122]. Artemisinin derivatives (artemether and artesunate) have a similar effect to PZQ, but they have a better killing effect on juvenile worms than adults [123], so the combination of the 2 drugs definitely has a better therapeutic effect [124]. It is currently hypothesized that artemisinin derivatives generate free radicals through heme-dependent reduction, which in turn cause lethal damage to schistosomes through the alkylation of parasitic proteins [42,125]. Surprisingly, the intestinal tracts of schistosomes are severely damaged after artemether treatment [126]. Another anti-schistosome drug candidate, dithiocarbamates, was recently reported to be nontoxic to human cells in experiments [127]. According to research findings, dithiocarbamates can damage the tegument, cause intestinal dilatation, inhibit worm spawning, and reduce their pair stability and viability [127]. Research on the schistosome vaccine is also a hot spot, and a systematic review has been made [9].

Killing or reduction in parasitic pathogenicity by structural destruction or inhibition of nutrient uptake proves to be a promising therapy. But only a few candidates have made it to human clinical trials. One of the reasons is the limitations of in vitro schistosome culture techniques. Schistosomes grow well during in vitro culture and can be grown from the early schistosomulum stage to the adult worm stage [128], but they have serious defects in reproduction. The inability of in vitro-cultured schistosomes to lay eggs, which are crucial for pathogenicity in human host, certainly hinders the development of drugs or vaccines. Another reason for this situation may be the limitations of animal models. For successful outcomes, experts suggested that vaccine should be tested on animal models preferably baboons [129].

Vaccine and drug development is time and resource intensive which could take decades for successful outcomes. Taking the funding limitations for parasitic research into account, many processes should be aforethought. In addition, data obtained through mouse-only model studies should be used with caution when designing clinical trial protocols [130]. In response to this global parasitic disease, laboratories working on schistosomes worldwide should be encouraged to work together to develop drugs and vaccines for the treatment and prevention of schistosomiasis, taking into account the parasitic structure, nutrient uptake, and their various life functions. Generally, we illustrated the structural basis and pathways of schistosome to obtain nutrients and summarized the related emerging targets. We also summarized several bottlenecks in this field in the hope of attracting professionals’ attention and promoting the application of strategies that combine nutrient blocking and structural disruption.
